# Marked Global Differences in Mortality in Male Patients with COVID-19: An Analysis of the CARDIO COVID 19–20 and WHF COVID-19 CVD Studies

**DOI:** 10.5334/gh.1403

**Published:** 2025-02-28

**Authors:** Juan Esteban Gómez-Mesa, Juan Pablo Arango-Ibanez, Pablo Perel, Dorairaj Prabhakaran, Hoover O. León-Giraldo, Alejandro Toro-Pedroza, Ricardo Enrique Larrea Gómez, César J. Herrera, Julián Lugo-Peña, Liliana Patricia Cárdenas Alaz, Victor Rossel, Daniel Sierra-Lara, Jessica Mercedes, Clara Inés Saldarriaga-Giraldo, María Juliana Rodríguez-González, Armando Alvarado, Juan Carlos Ortega, Miguel Quintana Da Silva, Kavita Singh, Karen Sliwa, On behalf of the CARDIO COVID 19–20 and WHF CVD COVID-19 research groups

**Affiliations:** 1 Departamento de Cardiología, Fundación Valle del Lili, Cali, Colombia; 2 Centro de Investigaciones Clínicas, Fundación Valle del Lili, Cali, Colombia; 3 Facultad de Ciencias de la Salud, Universidad Icesi, Cali, Colombia; 4 Department of Non-communicable Disease Epidemiology, London School of Hygiene & Tropical Medicine, World Heart Federation, Switzerland; 5 Public Health Foundation India, Centre for Chronic Disease Control, World Heart Federation, London School of Hygiene & Tropical Medicine, UK; 6 Departamento de Cardiología, Clínica Dávila, Santiago, Chile; 7 Departamento de Cardiología, Centros de Diagnóstico y Medicina Avanzada y de Conferencias Médicas y Telemedicina (CEDIMAT), Santo Domingo, Dominican Republic; 8 Departamento de Cardiología, Centro de Diagnóstico, Medicina Avanzada y Telemedicina (CEDIMAT), Santo Domingo, República Dominicana; 9 Departamento de Cardiología, Hospital Eugenio Espejo, Quito, Ecuador; 10 Departamento de Cardiología, Hospital del Salvador, Santiago de Chile, Chile; 11 Facultad de Medicina, Universidad de Chile, Santiago, Chile; 12 Departamento de Cardiología, Instituto Nacional de Cardiología Ignacio Chávez, Ciudad de México, México; 13 Departamento de Cardiología, Hospital Nacional San Rafael, Santa Tecla, El Salvador; 14 Departamento de Cardiología Clínica y Falla Cardiaca, Clínica CardioVID, Medellín, Colombia; 15 Departamento de Cardiología, LaCardio-Fundación Cardioinfantil, Bogotá, Colombia; 16 Hospital Especializado de Villa Nueva, Villa Nueva, Guatemala; 17 Departamento de Cardiología, Hospital Universitario Erasmo Meoz, Cúcuta, Colombia; 18 Departamento de Cardiología, Instituto Cardiovascular Sanatorio MIGONE, Asunción, Paraguay; 19 Public Health Foundation of India, Gurugram, Haryana, India; 20 Centre for Chronic Disease Control, New Delhi, India; 21 Heidelberg Institute of Global Health, University of Heidelberg, Heidelberg, Germany; 22 Cape Heart Institute, Department of Medicine & Cardiology, Groote Schuur Hospital, Faculty of Health Sciences, University of Cape Town, South Africa, World Heart Federation, Switzerland

**Keywords:** COVID-19, SARS-CoV-2 virus, men, male, mortality, global differences, Africa, America, Asia, Europe

## Abstract

**Background:**

COVID-19 has led to nearly seven million deaths and male sex has been reported as one of the main risk factors for mortality. Few studies have analyzed cohorts of male patients, especially in underrepresented regions in the medical literature, such as low and middle-income nations. To address this gap, we conducted large-scale, male-specific, multinational analyses, to improve understanding of factors associated with mortality in this high-risk population and global variations.

**Methods:**

This is a prospective, multicenter study that includes data from the CARDIO COVID-19–20 registry and the WHF COVID-19 CVD study. A multiple Poisson regression model was performed to evaluate differences in factors associated with in-hospital mortality among male COVID-19 patients across different regions.

**Results:**

We analyzed 4,899 hospitalized male COVID-19 patients from 32 countries: Africa (11.2%), the Americas (44.7%), Asia (33.8%), and Europe (10.2%). Median age was 59 years (IQR: 47–69), with 50.5% aged 40–64. ICU admission was 42.4%, and mortality was 19.2%, with marked regional differences (ranging from 6% in Europe to 26.9% in the Americas). Poisson regression showed age >80 years (aRR = 4.21) and IMV (aRR = 3.80) as the strongest factors associated with mortality. Other factors included diabetes, chronic kidney disease, myocarditis, and decompensated heart failure. Mortality risk was higher in Africa (aRR = 3.86), Asia (aRR = 2.72), and the Americas (aRR = 2.23) compared to Europe (p < 0.001). Anticoagulation/Antiplatelet therapy showed a potential correlation with survival.

**Conclusion:**

This study reflects the complexity of factors influencing COVID-19 mortality among male patients hospitalized with COVID-19, emphasizing global variability. The substantial differences in mortality noted across countries are likely due to differences in disease severity, comorbidities, clinical care, and health system factors. Age remains a primary risk factor, with older populations particularly vulnerable. Our findings underscore the need for targeted and tailored regional approaches to manage male COVID-19 patients.

## Background

The SARS-CoV-2 virus, responsible for Coronavirus Disease 2019 (COVID-19), triggered a pandemic that caused one of the most significant challenges faced by healthcare systems (1). As of August 2024, more than seven million deaths caused by COVID-19 have been reported (2). Most patients experienced a mild course; however, the associated mortality can be substantial in severe forms, with reports indicating general case-fatality rates of up to 10% (3). Mortality in patients with COVID-19 is highly dependent on risk factors such as advanced age and chronic comorbidities like diabetes mellitus (DM), obesity, and chronic kidney disease (CKD), among many others (4).

Male sex is a risk factor for higher COVID-19 severity, ICU admission rates, and mortality rates (5). This may stem from men’s tendency to delay seeking healthcare, higher rates of risky behaviors such as smoking and alcohol use, and greater prevalence of comorbidities such as cardiovascular disease and DM (6). Biological sex differences may also explain the mortality gap. For example, men tend to have a more intense inflammatory response than women when infected with COVID-19 (6,7). Together, these factors contribute to worse COVID-19 outcomes in men than women.

Regional disparities further complicate the interplay of risk factors for COVID-19 outcomes. Factors such as the timing and location of virus introduction, population density, hospital beds per 100,000 people, COVID-19 cases per 100,000 people, community mitigation measures, diagnostic testing capacity, and public health reporting practices have all been identified as influential determinants of mortality (8,9,10). Notably, infection fatality rates are roughly twice as high in developing countries compared to high-income countries, likely due to socioeconomic factors (11). These regional factors may interact with male-specific risks, amplifying the challenges faced by men in lower-resource settings. For example, limited healthcare access could exacerbate men’s delayed healthcare-seeking behaviors, while regional variations in the prevalence of comorbidities like obesity and diabetes may further compound the risk of severe outcomes.

Despite these insights, no reported studies have specifically examined the factors associated with mortality in a stratified group of male patients across different countries. Investigating these factors in a global context is critical, as it allows for identifying male-specific risks without the confounding or modifying effects of sex-based differences. The importance of this research persists, even with the decline in COVID-19 cases, as it provides vital insights for future health crisis preparedness and the implementation of strategies tailored to the needs of these populations. By understanding mortality-associated factors in male patients globally, we can better prepare for potential recurrences of COVID-19 and strengthen our responses to similar infectious diseases. To this end, we conducted a study evaluating mortality factors in hospitalized male patients with COVID-19 across multiple countries from different World Health Organization (WHO) regions.

## Methods

### Study design and setting

This observational, prospective, multicenter study utilized two existing databases of hospitalized COVID-19 patients: CARDIO COVID 19–20 (12) and WHF COVID-19 CVD and study (13). The first is a prospective study of patients diagnosed with COVID-19 who required in-hospital management between June 01, 2020, and June 30, 2021. This registry included 3,260 patients from 14 countries in Latin America. This study was coordinated by the Scientific Committee of the Council for Heart Failure and Pulmonary Hypertension (CIFACAH) of the Inter-American Society of Cardiology (SIAC).

The WHF COVID-19 and CVD study was a prospective cohort study that included 5,313 hospitalized patients between June 06, 2020, and September 15, 2021, from 40 hospitals across 23 countries in four continents. The World Heart Federation and Public Health Foundation of India coordinated this study.

**Figure 1:**
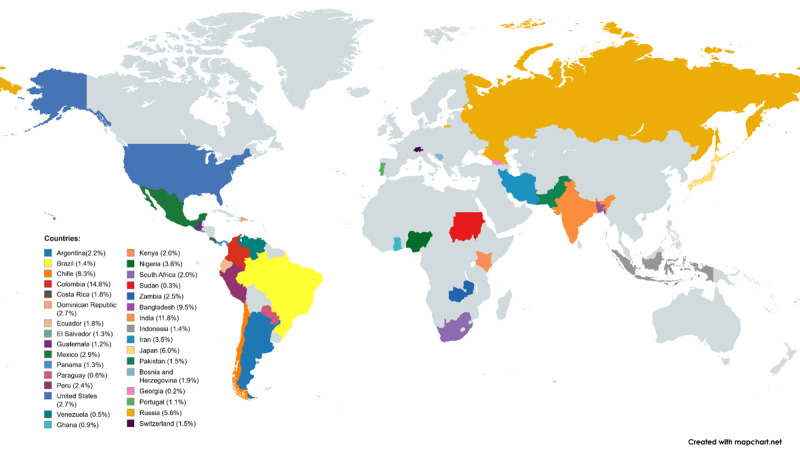
Participating countries and contribution to patient recruitment.

Figure 1 displays participating countries and their percentage of patient recruitment by participating hospital sites. It is important to note that Latin American countries contributed the majority of patients in the Americas. Although North American countries participated in patient recruitment, their representation was lower compared to Latin America.

The integration of the two studies was performed to ensure consistency in data. A detailed examination of the variable lists in both records was conducted to identify similar or equivalent variables while distinguishing exclusive variables unique to each record. An alignment of variables across both records was accomplished. Duplicated patients were found and excluded from the analysis. A standardization process of clinical parameter units was implemented to ensure data comparability and consistency.

### Study Participants

Male patients aged 18 years or older with a laboratory diagnosis of COVID-19 were included. Exclusion criteria for the CARDIO COVID 19–20 registry encompassed: patients hospitalized for less than 24 hours and those who died within 24 hours of consultation. Exclusion criteria for the WHF COVID-19 study were: patients without complete follow-up at 30 days, and those unlikely to stay at the participating center (e.g. transfers). For this sub-analysis, we excluded female patients.

### Statistical analysis

Exposure was hospitalization due to COVID-19. The primary outcome was in-hospital mortality. The distribution of continuous variables was assessed using the Kolmogorov-Smirnov test. Median and interquartile range (IQR) were used for non-normally distributed data. Qualitative data are displayed with absolute frequencies and percentages.

Univariate and multivariate analyses were conducted to calculate relative risks and their 95% confidence intervals (CI). For the effect-size multivariate analysis, we used a Poisson regression model with robust variance to account for the intrinsic variability of the study (data from multiple centers in different countries) and the high prevalence of the primary outcome. Variables were selected based on their statistical significance in the univariate analysis (p < 0.25) and their clinical relevance from previous literature. The influence of variables was assessed, and those lacking statistical significance were sequentially removed (backward elimination) to obtain the final model. Multicollinearity was evaluated using the generalized variance-inflation factor, and variables with a value greater than two were removed one by one.

Next, we included the continents (Africa, America, and Asia) as variables to evaluate potential differences in risks across the complete dataset. These variables were not included in the final model but were used to assess potential continental differences in risks after adjusting for multiple variables. Europe was used as the reference.

We then evaluated the model for each continent, resulting in five models (one global and one for each continent). This approach aimed to facilitate the comparison of factors associated with mortality across different regions. The statistical analysis was conducted using RStudio V.1.4.1717. Figures were created with Python version 3.13.1. MapChart was used to create a global map to highlight countries with participating institutions.

### Ethical considerations

The Comité de ética en Investigación Biomédica (Institutional Review Board) of the Fundación Valle del Lili in Cali, Colombia, approved this study. The WHF COVID-19 study received ethics approval from the University of Cape Town (South Africa), the Centre for Chronic Disease Control (India), and the Public Health Foundation (India). Each participating institution required ethics committee approval before patient recruitment. National regulatory clearances were also obtained for the WHF COVID-19 study. This study follows the guidelines of the Declaration of Helsinki.

In the WHF CVD study, informed consent was obtained following local ethical guidelines, either from participants or their legal representatives if they were unable to provide consent. In the CARDIO COVID Study, informed consent was not required due to its observational nature, but a validated consent form was used when mandated by institutional policies.

## Results

We included 4,899 male patients hospitalized with COVID-19, distributed across 32 countries spanning 4 continents. The patient distribution across the WHO region was: 551 patients (11.2%) from Africa, 2,188 patients (44.7%) from the Americas, 1,654 patients (33.8%) from Asia, and 506 patients from Europe (10.2%).

The overall median age was 59 years (IQR: 47–69), and most patients (n = 2,476, 50.5%) were aged between 40 and 64 years. The most common comorbidities included hypertension (n = 2,284, 46.6%), DM (n = 1,458, 29.8%), and obesity (n = 761, 23.4%). Data regarding demographics and comorbidities is shown in Table 1.

**Table 1:** Demographics and comorbidities by continent.


VARIABLE	OVERALL, n = 4899, (%)	AFRICA, n = 551, (%)	AMERICAS, n = 2188, (%)	ASIA, n = 1654, (%)	EUROPE, n = 506, (%)	p-VALUE

Age, years, median, IQR	59 (47–69)	55 (43–67)	60 (48–70)	58 (46–68)	60 (48–70)	<0.001

Age <40, years	679 (13.9%)	98 (17.8%)	240 (11%)	264 (16%)	77 (15.2%)	<0.001

Age 40–64, years	2476 (50.5%)	292 (53%)	1127 (51.5%)	819 (49.5%)	238 (47%)	<0.001

Age 65–79, years	1376 (28.1%)	130 (23.6%)	626 (28.6%)	483 (29.2%)	137 (27.1%)	<0.001

Age ≥80, years	368 (7.5%)	31 (5.6%)	195 (8.9%)	88 (5.3%)	54 (10.7%)	<0.001

Hypertension	2284 (46.6%)	269 (48.8%)	1021 (46.7%)	711 (43.0%)	283 (55.9%)	<0.001

Diabetes Mellitus	1458 (29.8%)	137 (24.9%)	586 (26.8%)	618 (37.4%)	117 (23.1%)	<0.001

Obesity	761 (23.4%)	66 (28%)	455 (31.1%)	114 (9.6%)	126 (34.3%)	<0.001

Chronic Kidney Disease	401 (8.2%)	37 (6.7%)	183 (8.4%)	113 (6.8%)	68 (13.4%)	<0.001

Coronary Artery Disease	552 (11.3%)	25 (4.5%)	183 (8.4%)	253 (15.3%)	91 (18%)	<0.001

Atrial Fibrillation	144 (2.9%)	3 (0.5%)	76 (3.5%)	19 (1.1%)	46 (9.1%)	<0.001

Heart Failure	271 (5.5%)	18 (3.3%)	121 (5.5%)	63 (3.8%)	69 (13.6%)	<0.001

Cancer	121 (2.5%)	3 (0.5%)	74 (3.4%)	19 (1.1%)	25 (4.9%)	<0.001

Smoking*	1201 (24.5%)	81 (14.7%)	396 (18.1%)	479 (29.0%)	245 (48.4%)	<0.001


IQR = Interquartile range. *Smoking History or Current Smoking Status.

**Figure 2:**
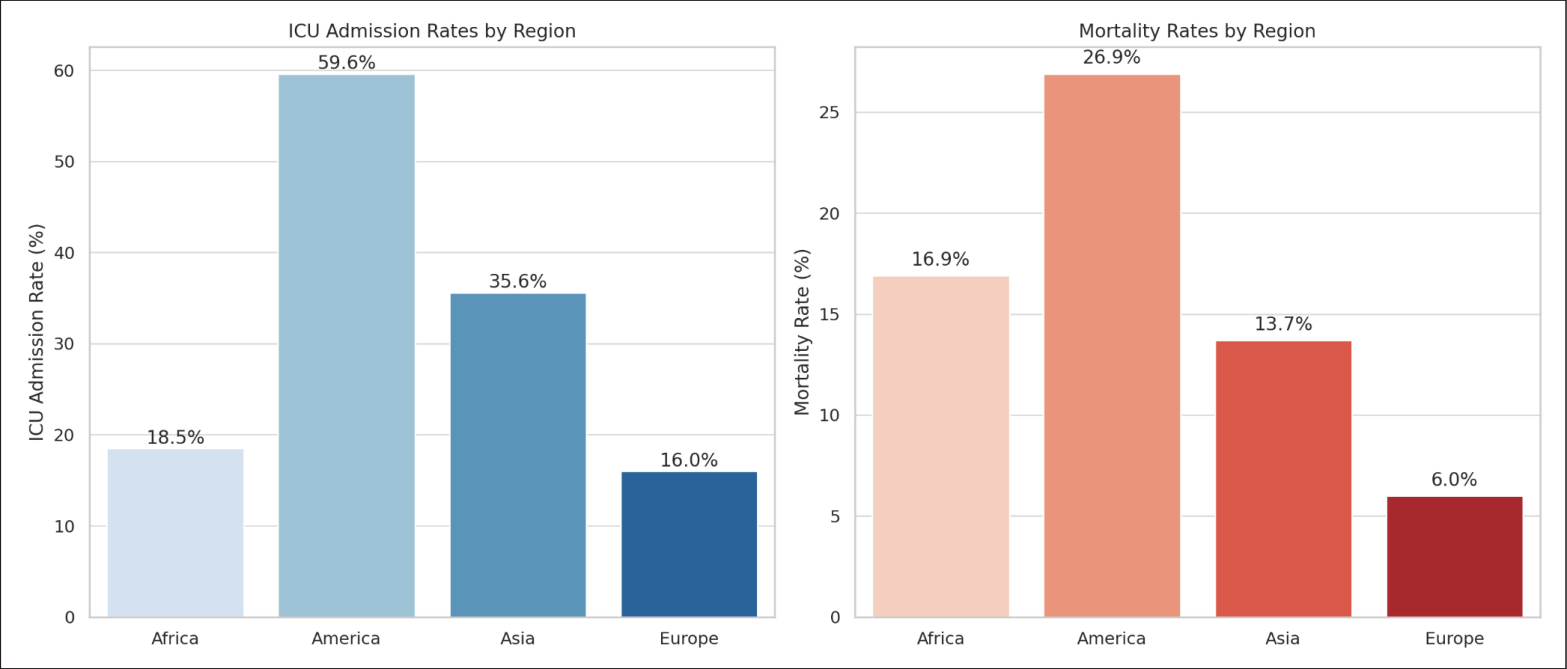
Intensive care unit admission rate and mortality rate by continent. ICU: Intensive care unit.

Table 2 shows the clinical characteristics of the patients including vital signs at admission, acute cardiovascular complications, pulmonary findings on chest imaging, medical treatment, and outcomes. Overall ICU admission was 42.4%, and mortality was 19.2%. Figure 2 represents mortality and ICU admission by continent.

**Table 2:** Clinical characteristics by continent.


VARIABLE	n FOR ANALYSIS	OVERALL, n (%)/MEDIAN (IQR)	AFRICA, n (%)/MEDIAN (IQR)	AMERICAS, n (%)/MEDIAN (IQR)	ASIA, n (%)/MEDIAN (IQR)	EUROPE, n (%)/MEDIAN (IQR)	p-VALUE

Vital signs at admission							

Respiratory rate (respirations per minute)	4050	22 (20–26)	24 (21–28)	23 (20–28)	22 (20–24)	20 (18–22)	<0.001

Heart rate (beats per minute)	4821	92 (80–105)	94 (81–105)	93 (81–107)	91 (80–104)	88 (80–99)	<0.001

Systolic blood pressure (mmHg)	4828	128 (115–140)	133 (121–148)	127 (113–140)	125 (114–140)	130 (120–140)	<0.001

Diastolic blood pressure (mmHg)	4829	79 (70–85)	83 (72–91)	75 (69–84)	80 (70–85)	80 (70–86)	<0.001

Temperature (Celcius)	4683	37 (36.5–38)	36.6 (36.2–37.1)	37 (36.4–37.9)	37.1 (36.6–38)	37.6 (37.0–38.2)	<0.001

Oxygen saturation (%)	4592	93 (88–96)	93 (88–96)	91 (85–95)	95 (92–97)	94 (91–96)	<0.001

Cardiovascular complications							

Arrhythmia	4899	299 (6.1%)	11 (2%)	216 (9.9%)	24 (1.5%)	48 (9.5%)	<0.001

Myocarditis	4899	48 (1%)	5 (0.9%)	27 (1.2%)	14 (0.8%)	2 (0.4%)	0.3

Pulmonary embolism	4899	156 (3.2%)	6 (1.1%)	98 (4.5%)	29 (1.8%)	23 (4.5%)	<0.001

Acute coronary syndrome	4899	113 (2.3%)	5 (0.9%)	69 (3.2%)	27 (1.6%)	12 (2.4%)	0.002

Acute heart failure	4899	286 (5.8%)	20 (3.6%)	183 (8.4%)	61 (3.7%)	22 (4.3%)	<0.001

Pulmonary findings							

Lung Infiltrates	3188*	2730 (85.6%)	127 (90.7%)	1790 (88.0%)	647 (80.8%)	166 (77.6%)	<0.001

Pleural effusion	3188*	288 (9%)	20 (14.3%)	215 (10.6%)	29 (3.6%)	24 (11.2%)	<0.001

In-hospital treatment							

Inotropes/Vasopressors	4899	795 (16.2%)	17 (3.1%)	685 (31.3%)	60 (3.6%)	33 (6.5%)	<0.001

Corticosteroid	4899	3438 (70.2%)	364 (66.1%)	1586 (72.5%)	1096 (66.3%)	392 (77.5%)	<0.001

Anticoagulant/Antiplatelet	4899	3346 (68.3%)	195 (35.4%)	1955 (89.4%)	814 (49.2%)	382 (75.5%)	<0.001

Invasive mechanical ventilation	4899	919 (18.8%)	26 (4.7%)	784 (35.8%)	74 (4.5%)	35 (6.9%)	<0.001

Clinical outcomes							

ICU admission	4899	2075 (42.4%)	102 (18.5%)	1303 (59.6%)	589 (35.6%)	81 (16%)	<0.001

In-hospital mortality	4899	934 (19.2%)	91 (16.9%)	587 (26.9%)	226 (13.7%)	30 (6%)	<0.001


ICU = Intensive care unit. IQR = Interquartile range. *Missing data due to unavailability of radiological imaging in 1,711 patients.

Using the World Health Organization data on hospital beds/10,000 population (14), we estimated the weighted mean value of this parameter for each continent, adjusting for the percentage contribution of patients from each participating country. We found that the mean number of beds per 10,000 population was 13.13 in Africa, 17.49 in the Americas, 31.98 in Asia, and 53.73 in Europe.

**Figure 3:**
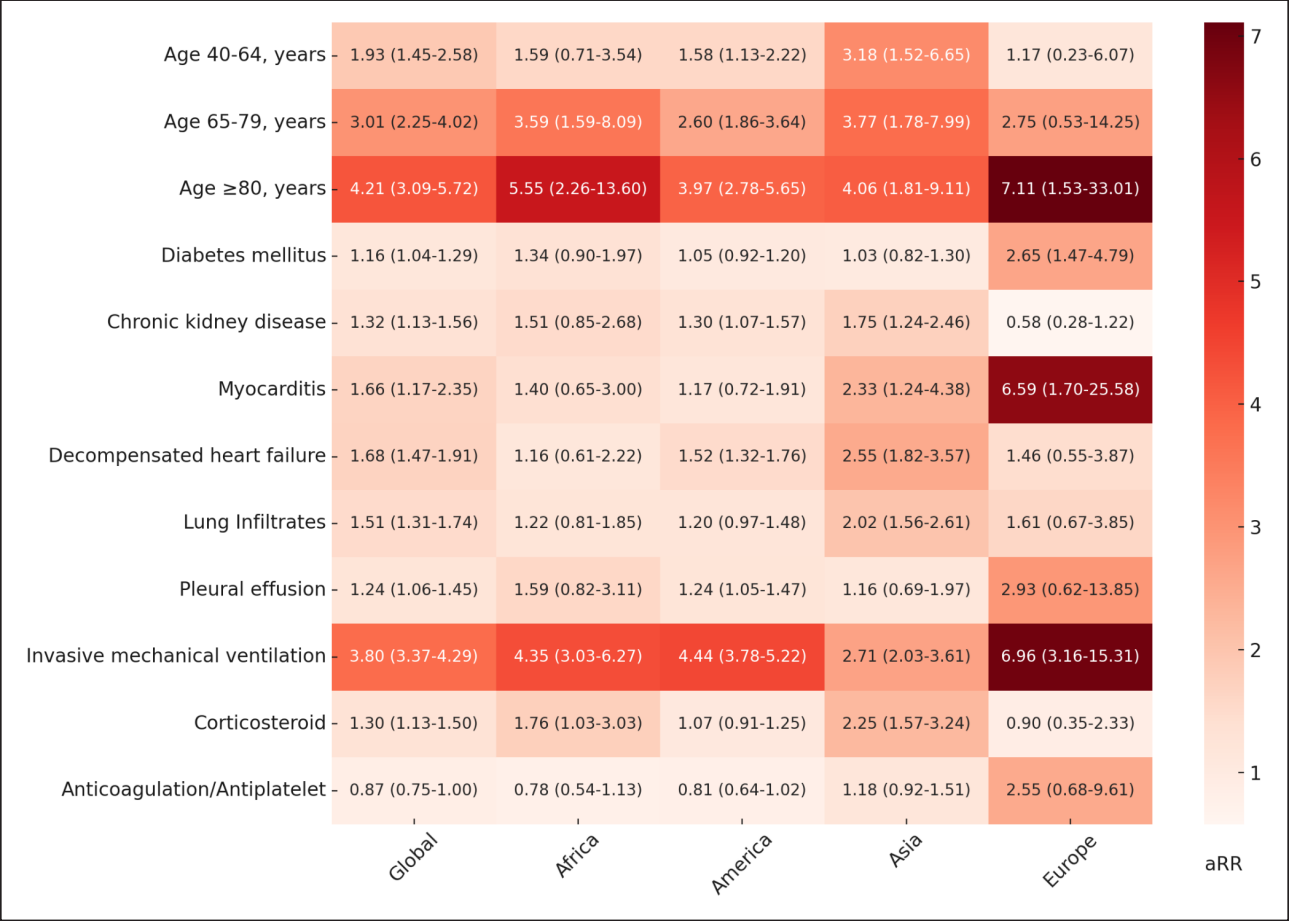
Heatmap of factors associated with in-hospital COVID-19 mortality by continent. aRR: adjusted relative risk. For each value, the aRR is presented along with its 95% confidence interval. In these statistical models, 4,873 (99.4%) patients were included in the ‘Global’ model, 537 in Africa, 2,180 in the Americas, 1,652 in Asia, and 504 in Europe.

Figure 3 represents five Poisson regression models with robust variance of factors associated with mortality. In the global model, the risk for mortality was higher for the three age groups in ascending order, a finding consistently seen in all continents. The biggest factor associated with mortality was an age greater than 80 years (aRR = 4.21; 95% CI: 3.09–5.72) followed by IMV (aRR = 3.80 95% CI: 3.37–4.29). The use of an anticoagulant/antiplatelet had an effect associated with decreased mortality, although the 95% CI included the unit (P = 0.058). This variable was still included in the model due to its clinical significance and the prominent effect direction it demonstrated.

We conducted an exploratory analysis to evaluate the differences between continents in mortality; we added Africa, the Americas, and Asia as independent variables to the final global model and used Europe as the reference. We found the following adjusted relative risks (aRR): Africa 3.86 (CI 95 2.67–5.57, p < 0.001), Americas 2.23 (CI 95 1.58–3.15, p < 0.001), and Asia 2.72 (CI 95 1.92–3.84, p < 0.001). Including the continents in the model did not affect the magnitude of the effects of all other variables by more than 10% except for anticoagulation/antiplatelet, which showed an aRR of 1.01 (CI 0.86–1.19, p = 0.893).

In Africa, IMV was associated with higher mortality (aRR = 4.35; 95% CI: 3.03–6.27), along with corticosteroid use (aRR = 1.76; 95% CI = 1.03–3.03). As observed in Europe and Asia, the highest aRR is seen in patients over 80 years old.

In Americas, a high risk of mortality was observed in patients with CKD (aRR = 1.30; 95% CI: 1.07–1.57); DHF (aRR = 1.52; 95% CI: 1.32–1.76); pleural effusion (aRR = 1.24; 95% CI: 1.05–1.47); and IMV (aRR = 4.44; 95% CI: 3.78–5.22). No significance was observed in DM, myocarditis, lung infiltrates, corticosteroids, and anticoagulation/antiplatelet use. The variable with the highest risk was IMV, followed by age over 80 years.

In Asia, factors associated with mortality included CKD (aRR = 1.75; 95% CI: 1.24–2.46); the presence of myocarditis (aRR = 2.33; 95% CI: 1.24–4.38); DHF (aRR = 2.55; 95% CI: 1.82–3.57); lung infiltrates (aRR = 2.02; 95% CI: 1.56–2.61); IMV (aRR = 2.71; 95% CI: 2.03–3.61) and use of steroids (aRR = 2.25; 95% CI: 1.57–3.24). The variables most strongly associated with mortality were ages over 80 years and ages between 65 and 79 years.

In Europe, a significant risk of mortality was identified in patients aged over 80 years (aRR = 7.11; 95% CI: 1.53–33.01), and a smaller effect was observed in the two other age groups. Other factors associated with mortality included DM (aRR = 2.65; 95% CI: 1.47–4.79), myocarditis (aRR = 6.59; 95% CI: 1.70–25.58), and IMV (aRR = 6.96; 95% CI: 3.16–15.31). The variables most strongly associated with mortality were ages over 80 years, followed closely by IMV and myocarditis. The use of anticoagulant/antiplatelet presented an aRR and CI consistent with a potential effect associated with mortality, in contrast to the findings of the global model.

Table 3 (Supplementary Material) presents a logistic regression model of factors associated with in-hospital COVID-19 mortality by continent for sensitivity analysis. Variables were selected using the same approach described in the Statistical Analysis section of this manuscript. This analysis yielded similar patterns to those observed in the Poisson model with robust variance.

## Discussion

This study presents an analysis of the factors associated with COVID-19 in-hospital mortality among men from Africa, the Americas, Asia, and Europe. To the best of our knowledge, this is the first study conducting a continent-stratified regression analysis on male patients hospitalized with COVID-19. The most notable finding is the high mortality in the Americas (predominantly Latin America) and, to a lesser extent, in Africa, compared to Asia and Europe. Additionally, we found an association with mortality in factors including age, comorbidities (DM, CKD), cardiovascular complications (myocarditis, DHF), lung infiltrates, pleural effusion, corticosteroids, and IMV.

Overall mortality in our study (19.2%) is consistent with a meta-analysis that found a mortality rate of 16% (95% CI 12–21, I^2^ = 100%) in hospitalized COVID-19 patients (15). However, the mortality in the Americas (26.9%) was substantially higher. Age does not appear to drive the global differences in mortality in our study, as the continents with the highest (Americas) and lowest (Europe) mortality rates had a comparable proportion of patients over 65 years (37.5% vs 37.8%, respectively). The substantial differences in mortality cannot be adequately explained by the impact of comorbidities, such as DM and CKD, the only comorbidities associated with mortality in our model. In the regression analysis, they showed only modest associations in terms of magnitude and diverse patterns of aRR across continents. The vaccination rate among patients in the WHF COVID-19 registry was substantially low, and there were no vaccinated patients in the CARDIO COVID 19–20 registry; thus vaccination is also not a potential explanation (16,17). These findings indicate that other unaccounted variables may have had a higher impact on mortality differences across continents. This hypothesis is further supported by our analysis incorporating continent as a variable in the final global model, which revealed that Africa, the Americas, and Asia were independently associated with increased mortality risk compared to Europe, even after adjusting for other factors.

The higher ICU admission rates in the Americas and Africa suggest a greater proportion of patients with severe illness in our cohort for these regions, which could partially explain the difference in mortality (18,19). This is also supported by data showing more frequent abnormal vital signs at admission, lung infiltrates, cardiovascular complications, and the need for IMV in these regions. Additionally, differences in the number of hospital beds/10,000 population may partially explain this, as it has been identified as a country-level predictor of COVID-19 mortality (10). The weighted average of beds/10,000 habitants was substantially lower in Africa and the Americas compared with Asia and Europe. Other factors that could explain the differences in mortality include comorbidity burden, quality of care, hospital resources, and treatment protocols (20,21,22). Thus, regions with stronger healthcare systems may be more effective in managing pandemics and reducing mortality rates.

Previous studies have explored factors associated with mortality in men. One study included 294 hospitalized male patients and found that pulmonary disease and connective tissue disease were significant factors, along with age over 70 years, quick Sequential Organ Failure Assessment (qSOFA) score, and laboratory markers such as C-reactive protein, lymphocytopenia, and thrombocytopenia (23). Another study, which included 2,757 male patients, found that age (>64) and hypoxia (oxygen saturation <92%) were associated with increased mortality. No significant effects were observed in different ethnic groups (e.g., Hispanic, Asian, non-Hispanic White) or various comorbidities (e.g., DM, CDK, heart failure) (24).

In this study, age was the primary factor linked to mortality, demonstrating a direct proportionality across age ranges (40–64, 65–79, ≥80 years). Individuals aged 80 years or older exhibited the highest risks. These findings align consistently with those of extensive studies (4,25). However, one key observation in our study is that while Europe had the largest proportion of patients aged ≥80, the Americas exhibited higher overall mortality. This suggests that although age is a significant factor in mortality, many other factors also play crucial roles in determining outcomes. CKD and DM were associated with in-hospital mortality, although notable differences can be seen across continents; these diseases are well-established major risk factors for COVID-19 mortality in the literature (4,18,26,27). Interestingly, we found that CKD had a non-significant effect in Europe, which aligns with results from a meta-analysis and is thought to be related to lifestyle or treatment modalities (28). The magnitude of its risk is similar to the one reported in a study incorporating multiple systematic reviews (27). Notably, Europe exhibited the highest risk associated with DM, despite having the lowest DM prevalence among all continents. Regional differences in how comorbidities affect COVID-19 outcomes likely stem from variations in healthcare, population traits, pandemic factors, and statistical power, among other factors.

The presence of pulmonary infiltrates in chest imaging was associated with increased mortality in our cohort. Previous research has established a correlation between lung infiltrates and severe COVID-19. This suggests that extensive pulmonary involvement, as indicated by this imaging finding, is linked to increased disease severity in COVID-19 patients (29,30). Pleural effusion was found to be associated with mortality, being consistent with a meta-analysis, including 6,234 patients, which found that this finding is associated with both severity and mortality (31).

Regarding cardiac complications during hospitalization, we found evidence of myocarditis as an important factor for mortality, especially in Asia and Europe. This complication has received attention given its association with both the virus and vaccines and has been shown to have a higher impact on men than on women (32,33). DHF was associated with mortality; this was also found in a large study that included 42,225 patients with DHF and COVID-19 (34).

The use of corticosteroids was associated with higher mortality. Corticosteroids have played a critical role in the treatment of COVID-19, with multiple early studies highlighting their potential to reduce mortality (35,36). However, their effectiveness appears to be modified by the severity of the disease. A meta-analysis, which included five randomized trials and one propensity score-matched study, suggested that corticosteroids might increase the risk of mortality in COVID-19 patients who are not receiving oxygen therapy (37). Our findings likely result from a combination of factors, including indication bias, in which corticosteroids were more frequently prescribed to patients with greater disease severity, and a potential true association with increased mortality. During the early pandemic, corticosteroids were widely used among hospitalized patients, as seen in this study, many of whom did not have severe disease.

We found a trend toward a protective effect with the use of anticoagulant/antiplatelet therapy. Evidence supports the decrease in mortality associated with therapeutic anticoagulation in moderate cases of COVID-19 (38). On the other hand, the use of antiplatelet therapies has inconsistent evidence on decreasing mortality (39). However, a recent propensity-scored-match cohort study demonstrated that antiplatelet therapy before COVID-19 decreases mortality (40). We evidenced differences in the effect direction in our study when analyzing the models by region: while an effect associated with decreased mortality was seen in the Americas and Africa, Europe, and Asia showed a trend towards higher risk. This suggests that other variables could have confounded the potential protective effects associated with using anticoagulation/antiplatelet.

Lastly, IMV was found to be consistently associated with mortality in all continents. Its use is highly related to COVID-19 severity and has been described as one of the leading factors associated with mortality in patients with severe disease (41). The global differences in the effects of the mentioned interventions could be explained by distinct prescription policies, and medications used, among other not accounted factors.

## Strengths/Limitations

The strengths of this study include its considerably large sample size, geographic representation, and inclusion of patients recruited from underrepresented countries of Sub-Saharan Africa, Asia, and Latin America. Additionally, it included a significant number of male patients from low- and middle-income countries, where there is limited information on COVID-19 health outcomes (42).

One key limitation of this study is the exclusion of patients with short hospital stays or those who passed away within 24 hours. This exclusion may have omitted critically severe cases, potentially introducing bias into the assessment of mortality risk. Therefore, our findings should be interpreted with caution and are most applicable to patients who do not meet these exclusion criteria. Another limitation of this study is the limited number of patients recruited from Europe and Africa. Our findings cannot be generalized to entire continents due to the underrepresentation or absence of certain countries within some regions. Nevertheless, we believe that stratifying data by continent offers valuable insights into global differences evidenced in our study.

Some variables had missing data that were not available in the electronic health records (e.g., radiological studies) and we did not perform imputation of missing data. Moreover, since a considerable amount of data comes from unvaccinated individuals given the early stage of the COVID-19 pandemic during which the patients were recruited, COVID-19 vaccination may alter the effect of risk factors regarding COVID-19 mortality in patients (43). Another limitation is the creation of composite variables (e.g. anticoagulant/antiplatelet), making it challenging to draw specific conclusions about one without the other. Additional studies with greater representation from all continents are needed to provide more robust and comprehensive conclusions regarding the aim of our research.

## Conclusions

This study reflects the complexity of factors influencing COVID-19 mortality in male patients, with a distinct global variability observed. The data reveal that age remains a primary risk factor, with older populations particularly vulnerable. Differences regarding comorbidities demonstrate varying degrees of influence across continents, highlighting the possible role of healthcare care and other environmental factors. This study highlights massive disparities in mortality across continents, especially affecting Hispanics and African men, which are likely to be highly influenced by socio-economic determinants and health system factors. The diverse findings concerning factors for mortality in male patients from various continents emphasize the need for region-specific strategies in patient management and further research to elucidate regional disparities.

## Supplementary Material

**Table 3:** Logistic regression analysis of factors associated with in-hospital COVID-19 mortality by continent.


VARIABLE	OVERALL, n = 4873, aOR (95% CI)	AFRICA, n = 537, aOR (95% CI)	AMERICAS, n = 2180, aOR (95% CI)	ASIA, n = 1652, aOR (95% CI)	EUROPE, n = 504, aOR (95% CI)

Age 40–64, years	2.22 (1.56–3.22, p < 0.001)	1.89 (0.74–5.70, p = 0.212)	1.87 (1.18–3.05, p = 0.009)	3.29 (1.57–8.07, p = 0.004)	1.13 (0.15–24.36, p = 0.917)

Age 65–79, years	4.56 (3.18–6.68, p < 0.001)	5.90 (2.25–18.26, p = 0.001)	4.83 (3.01–7.98, p < 0.001)	4.20 (1.97–10.41, p = 0.001)	3.18 (0.47–66.34, p = 0.318)

Age ≥80, years	7.60 (5.02–11.70, p < 0.001)	10.76 (3.33–38.78, p < 0.001)	10.95 (6.30–19.48, p < 0.001)	3.98 (1.56–11.11, p = 0.005)	10.63 (1.45–232.01, p = 0.048)

Diabetes Mellitus	1.24 (1.03–1.48, p = 0.019)	1.66 (0.94–2.89, p = 0.075)	1.05 (0.81–1.36, p = 0.701)	1.07 (0.77–1.48, p = 0.684)	3.38 (1.37–8.52, p = 0.008)

Chronic Kidney Disease	1.65 (1.26–2.17, p < 0.001)	1.63 (0.68–3.66, p = 0.253)	1.75 (1.18–2.60, p = 0.006)	2.43 (1.45–3.99, p = 0.001)	0.47 (0.14–1.41, p = 0.201)

Myocarditis	2.67 (1.34–5.30, p = 0.005)	2.67 (1.34–5.30, p = 0.005)	1.04 (0.40–2.64, p = 0.940)	13.70 (3.72–59.18, p < 0.001)	14.01 (0.37–591.44, p = 0.124)

Decompensated heart failure	3.23 (2.41–4.32, p < 0.001)	1.19 (0.36–3.68, p = 0.763)	3.00 (2.06–4.38, p < 0.001)	6.76 (3.66–12.56, p < 0.001)	1.71 (0.44–6.10, p = 0.422)

Lung infiltrates	1.71 (1.41–2.08, p < 0.001)	1.20 (0.62–2.27, p = 0.585)	1.20 (0.62–2.27, p = 0.585)	2.46 (1.78–3.41, p < 0.001)	1.75 (0.66–4.61, p = 0.258)

Pleural effusion	1.49 (1.11–2.00, p = 0.008)	2.10 (0.64–6.75, p = 0.212)	1.58 (1.11–2.23, p = 0.010)	1.15 (0.42–2.94, p = 0.770)	3.59 (0.72–15.70, p = 0.100)

Invasive mechanical ventilation	8.42 (6.98–10.20, p < 0.001)	18.32 (6.75–55.86, p < 0.001)	11.15 (8.68–14.42, p < 0.001)	6.59 (3.88–11.25, p < 0.001)	12.09 (4.26–35.26, p < 0.001)

Corticosteroid	1.53 (1.25–1.88, p < 0.001)	2.09 (1.10–4.14, p = 0.028)	1.16 (0.89–1.53, p = 0.272)	3.00 (1.94–4.80, p < 0.001)	0.85 (0.26–3.18, p = 0.794)

Anticoagulation/Antiplatelet	0.74 (0.60–0.91, p = 0.004)	0.68 (0.37–1.22, p = 0.202)	0.64 (0.43–0.96, p = 0.028)	1.16 (0.83–1.61, p = 0.393)	2.75 (0.67–15.04, p = 0.192)


aORR = Adjusted odds ratio. CI = Confidence interval. First, candidate variables were assessed using univariate analysis. Variables with a p < 0.25 or considered clinically significant were selected for evaluation in the logistic regression model. A backward selection method was then applied to determine the final model for all participants. Finally, this analysis was stratified by continent.

## Data Availability

The data that support the findings of this study are available from the corresponding author upon reasonable request.

## Abbreviations

ACS: Acute coronary syndrome

aRR: Adjusted relative risk

CI: Confidence interval

COVID-19: Coronavirus Disease 2019

CKD: Chronic kidney disease

DHF: Decompensated heart failure

DM: Diabetes mellitus

qSOFA: Sequential organ failure assessment

ICU: Intensive care unit

IMV: Invasive mechanical ventilation

IQR: Interquartile range

PE: Pulmonary embolism

RR: Relative risk

SBP: Systolic blood pressure

WHF: World Heart Federation
